# It takes patience and persistence to get negative feedback about patients’ experiences: a secondary analysis of national inpatient survey data

**DOI:** 10.1186/1472-6963-14-153

**Published:** 2014-04-04

**Authors:** David N Barron, Elizabeth West, Rachel Reeves, Denise Hawkes

**Affiliations:** 1 Saïd Business School and Jesus College, University of Oxford, Park End Street, Oxford OX1 7HP, UK; 2 Mary Seacole Building, School of Health and Social Care, University of Greenwich, Avery Hill Road, Eltham, London SE9 2UG, UK; 3 School of Health and Social Care, University of Greenwich, Avery Hill Road, Eltham, London, UK; 4 Doctoral School at the Institute of Education, University of London, London, UK

**Keywords:** Patient satisfaction/statistics and numerical data, Hospitals/standards, Health care surveys/methods, Bias (epidemiology), Questionnaires

## Abstract

**Background:**

Patient experience surveys are increasingly used to gain information about the quality of healthcare. This paper investigates whether patients who respond before and after reminders to a large national survey of inpatient experience differ in systematic ways in how they evaluate the care they received.

**Methods:**

The English national inpatient survey of 2009 obtained data from just under 70,000 patients. We used ordinal logistic regression to analyse their evaluations of the quality of their care in relation to whether or not they had received a reminder before they responded.

**Results:**

33% of patients responded after the first questionnaire, a further 9% after the first reminder, and a further 10% after the second reminder. Evaluations were less positive among people who responded only after a reminder and lower still among those who needed a second reminder.

**Conclusions:**

Quality improvement efforts depend on having accurate data and negative evaluations of care received in healthcare settings are particularly valuable. This study shows that there is a relationship between the time taken to respond and patients’ evaluations of the care they received, with early responders being more likely to give positive evaluations. This suggests that bias towards positive evaluations could be introduced if the time allowed for patients to respond is truncated or if reminders are omitted.

## Background

Concerns about quality of healthcare have led to a proliferation of patient experience surveys. The national patient survey programme for England was first proposed in The National Health Service: Modern, Dependable [1] as a way of assessing patients’ experiences of care and how they change over time. The surveys were part of a more general commitment to make the National Health Service (NHS) more responsive to patients. The reasoning was—and still is—that if hospitals are given information about how patients evaluate the quality of the care they received, managers and clinicians will be able to respond to any identified shortcomings, leading to improvements in the quality of care. The surveys are a potentially important resource for NHS Trusts as they provide detailed information on experiences of care from probability samples of recent patients. However, their usefulness depends on the representativeness of those who respond.

The first hospital-based national survey of adult inpatients was reported in 2002 [2] and the survey has been repeated almost every year since then under a programme centrally monitored by the Care Quality Commission (CQC). Each NHS Trust in England is asked to conduct a postal survey of 850 consecutively-discharged recent inpatients. They may conduct the survey themselves or use a CQC-approved survey contractor, but all Trusts are required to adopt a standard methodology that attempts to maximise response rates by making up to three attempts to contact patients. The initial questionnaire is sent, followed by a reminder letter to non-responders around 21 days later, and then a second reminder with a duplicate questionnaire is sent to those who have still not responded after a further 21 days. Postage-free return envelopes are included with the first mailing and second reminder [3]. Each year, approximately 160 English acute NHS trusts participate in the national inpatient survey, and the results of their question scores are published by the CQC. Currently, each Trust’s survey scores are re-weighted to adjust for differences among trusts in responders’ age, sex and route of admission (planned or emergency). Each responder’s weight is calculated by dividing the proportion of respondents in the national data set for that year in their age/sex/admission route group by the Trust’s proportion. An upper limit for the weight is set at 5.

In 2009, the response rate for the annual inpatient survey was 52%. Response to the first mailing without the need for a reminder was 41%, and a further 11% were received after the reminder and second questionnaire had been dispatched. This raises the important question of whether the 11% of patients who responded after reminders differed in some systematic way from those who responded at the first invitation. If there are systematic differences, this suggests that closing the survey too soon after the first questionnaire and/or failing to send out reminders would have led to bias in measurements of patients’ experiences in hospitals in the NHS.

A systematic review into methods of increasing response rates to health surveys, [4] citing studies going back to 1921, found that a second and third mailings typically attracted further responses from 12% and 10% of the original sample, respectively, although these averages masked considerable variability. More recently, Nakash et al. [5] found that “more intense follow-up” increased response rates, although the different methods used by the studies (including telephone calls in one case) reduced its generalisability. A larger review of randomised controlled trials by Edwards et al. [6] found evidence for the effectiveness of follow-up contact, with the odds of response after follow-up being 1.35 (95% CI 1.18 to 1.55).

These studies suggest, then, that the use of repeat mailings, and sending a second copy of the questionnaire, are likely to increase response rates. However, that in itself does not demonstrate that response bias is reduced: questionnaire responses received after follow-up may not be systematically different from those received in response to the initial mailing. Mazor et al. [7] found a positive correlation between response rates to a survey about patient satisfaction with individual physicians and the physicians’ patient satisfaction scores—that is, more satisfied patients were more likely to respond. In a simulation study, they then showed that non-response bias would most likely lead to patient satisfaction being overestimated. Further, as they were dealing with data about patient satisfaction with individual physicians, they were able to conclude that the scores for the physicians with whom the patients were least satisfied would have the greatest magnitude of error.

Evidence of systematic differences between responders and non-responders is difficult to obtain, but some studies have shown that early and late responders to mail surveys sometimes differ. For example, one study of responders to a US patient satisfaction survey that involved nearly 20,000 patients in 76 hospitals [8] found significant differences between the first 30% of responders and the remainder of responders on nine out of thirteen scales. Similarly, Perneger et al. [9] showed that early responders reported significantly fewer problems with the healthcare they received than late responders or non-responders. In Norway, Bjertnaes conducted a national study of 10,912 recently-discharged patients based on a survey with important similarities to NHS national inpatient survey, which is the focus of this paper [10]. He found that satisfaction on five of the six reported patient satisfaction scales decreased as *response time* (the time it took patients to return questionnaires after they had been received) increased. More recently, Hutchings et al. [11] compared early and late responders to a large (n ≈ 80,000) UK survey of patient reported outcomes after four surgical procedures. After controlling for a range of variables previously found to be associated with non-response, including age, ethnicity, deprivation and health status [12], they found that late responders were slightly more likely to report poorer outcomes. These results are consistent with a number of other studies that have found an association between late response and patients’ tendency to report poorer clinical outcomes [9,13-16]. In summary then, the balance of evidence seems to suggest that there is a difference between early and late responders with the latter being less satisfied with their care or with the clinical outcomes of their treatment.

The evidence for the differences between initial and post-reminder responders is less clear. Yessis and Rathert [17] have suggested that reminders are important as they found that patients responding to a reminder were significantly less satisfied than were initial respondents. However, other researchers have found no significant difference between initial respondents and those who required several reminders and follow-ups to obtain a response [18]. Therefore, it is important that we investigate whether there is indeed a difference between initial and post follow-up responders in this survey.

It is also possible to go beyond seeing using repeated mailings as a way of increasing response rate. Some authors have suggested that people who respond later to mail surveys be treated as proxies for people who do not respond at all. Halbesleben and Whitman [19] explain that “[t]he logic behind this approach is based on a process called the continuum of resistance, which suggests that each subsequent wave of participants demonstrates greater resistance in completing the survey. By this logic, one could use the last people to respond (thus, the most difficult to obtain) as proxies for nonrespondents, as they are closest to nonrespondents on the continuum of resistance. Thus, we can compare the last group to respond with the others in the survey to examine potential differences that might approximate nonresponse bias”. (p. 11).

This study seeks to add to the available evidence by using a large sample, a large number of hospitals and a single mode of data collection. The key question is whether later respondents—and in particular those who respond to reminders—differ systematically from those who respond quickly. In this paper, we test whether there are significant differences between early and late responders, examine the relationship to reminders and to explore the possibility of using data from late responders as a proxy for non-responders.

More formally, the research questions are:

1. Is there an association between whether people are early or late respondents to the survey and their evaluations of the quality of the care they received?

2. Is the use of reminders an effective way of reducing non-response bias in survey-based estimates of patients’ evaluation of the quality of their care?

3. Can data from late responders be used as a way of estimating the effect of non-response bias?

## Methods

### National inpatient survey data

This study uses the data from the Care Quality Commission’s (CQC’s) 2009 English national inpatient survey. Annually, these data are archived in the UK Data Archive, but do not include questionnaire return dates. In addition, Picker Institute Europe, who collate and clean the data for the CQC, supplied the questionnaire return dates for the purposes of conducting this study, as agreed by the CQC. Further details of the sampling and survey methods have been described elsewhere [20]. For the 2009 survey, questionnaires were sent to a total of 137,360 recently discharged inpatients, of whom 69,348 returned usable responses. Excluding 1,831 undelivered questionnaires and 2,069 deceased patients, this corresponds to a response rate of 52%.

### The questionnaire

The questionnaire used in the Inpatient Survey asks patients to evaluate their care with reference to: access to information, hospital cleanliness, communications with clinical staff, responsiveness of hospital staff, pain management, co-ordination of care, information on discharge and relationships among clinicians. The questions are purposely designed to facilitate quality improvements by providing actionable feedback to healthcare professionals by asking patients to report *what happened* to them regarding specific aspects of their care episode, rather than eliciting general satisfaction ratings [21]. Therefore, for this study, we did not use a composite score but used five of the questions as dependent variables to measure patient satisfaction.

1. “Overall how would you rate the care you received?” Responses are “Excellent”, “Very good”, “Other”.

2. “In your opinion, how clean was the hospital room or ward that you were in?” Responses are “Very Clean”, “Fairly Clean”, “Other”.

3. “Did you have confidence and trust in the doctors treating you?” Responses are “Yes, always”, “Yes, sometimes”, “No”.

4. “Did you have confidence and trust in the nurses treating you?” Responses are: “Yes, always”, “Yes, sometimes”, “No”.

5. “How many minutes after you used the call button did it usually take before you got the help you needed?” Responses are: “0 minutes/right away”, “1-2 minutes”, “3-5 minutes”, “More than 5 minutes”, “I never got help when I used the call button”.

The frequencies of responses to these questions are shown in Table 1. These questions were chosen to represent, in addition to an overall rating of care, a measure of satisfaction with the physical condition of the hospital, measures of satisfaction with the two main health care professions and, in the case of the call button response time question, a measure of a more concrete aspect of nursing care.

**Table 1:** Responses to the care satisfaction questions

**Overall, how would you rate the care you received?**	**Excellent**	**Very good**	**Good, Fair** ***or*** **Poor**	
	30,038	23,228	13,880	
	(44.7%)	(34.6%)	(20.7%)	
**In your opinion, how clean was the hospital room or ward that you were in?**	**Very clean**	**Fairly clean**	**Not very clean** ***or*** **Not at all clean**	
	44,256	21,579	2,510	
	(64.7%)	(31.6%)	(3.7%)	
**Did you have confidence and trust in the doctors treating you?**	**Yes, always**	**Yes, sometimes**	**No**	
	55,031	11,116	2,124	
	(80.6%)	(16.3%)	(3.1%)	
**Did you have confidence and trust in the nurses treating you?**	**Yes, always**	**Yes, sometimes**	**No**	
	50,699	15,249	2,225	
	(74.4%)	(22.4%)	(3.3%)	
**How many minutes after you used the call button did it usually take before you got the help you needed?**	**0 minutes**	**1-2 minutes**	**3-5 minutes**	**I never got help**
	6,387	15,635	11,541	6,114
	(15.8%)	(38.8%)	(28.6%)	(15.2%)

### Statistical analysis

The main explanatory variable was whether a response was received without a reminder, following the first reminder, or following the second reminder. We also controlled for other factors that previous research has suggested may be associated with satisfaction with care. A systematic review [22] of all the published research outputs produced using the patient survey data showed that several patient characteristics are associated with their evaluation of care. In this study therefore we control for these factors including age, sex, length of stay in hospital, and whether the person was admitted as an emergency or not. Analysis was performed using ordinal logistic regression [23]. We used Stata 12 to perform the analysis, obtaining robust standard errors that control for the clustering of observations within Trusts [24].

## Results

**Figure 1:**
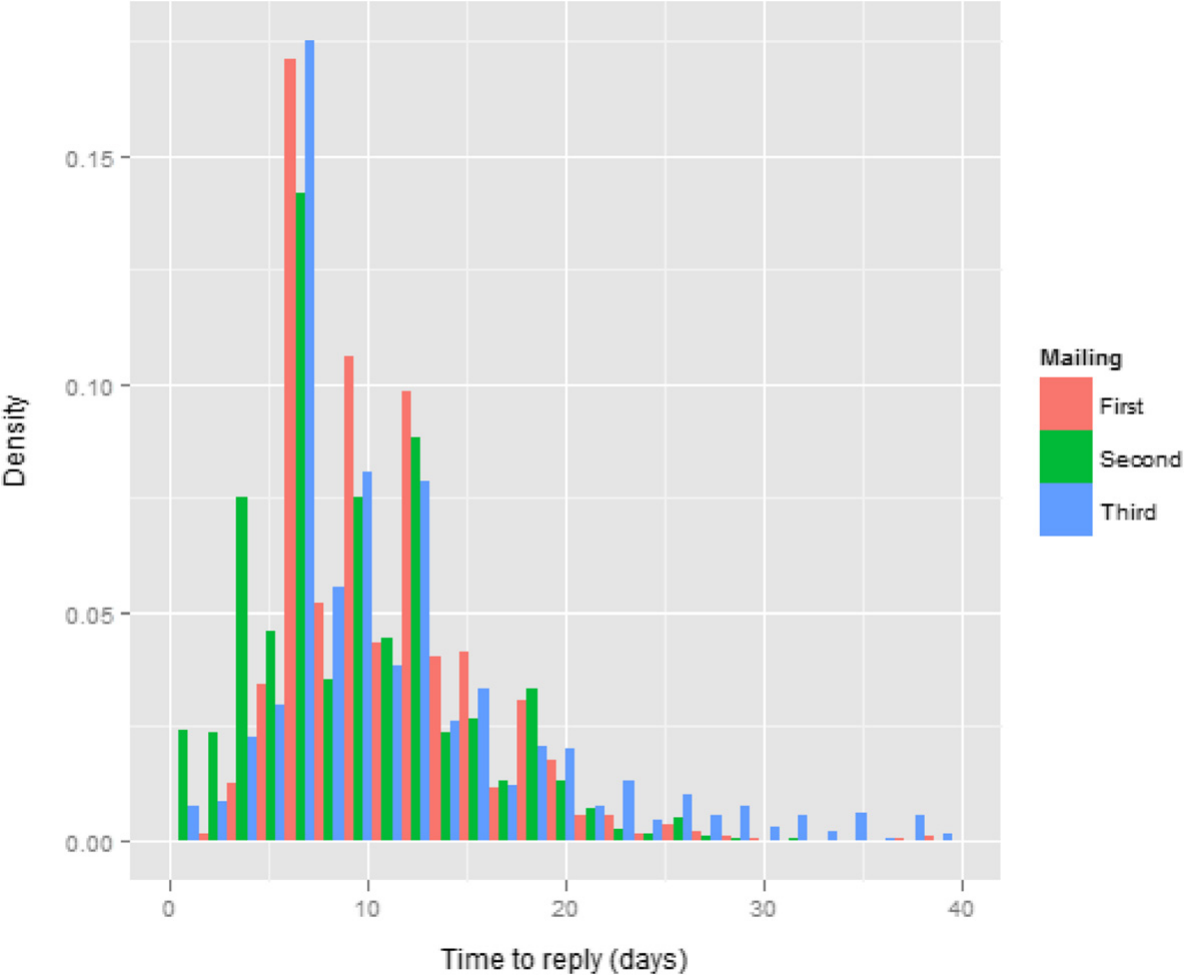
Bar chart showing the distribution of the length of time it took to receive a reply following the dispatch of a questionnaire or reminder.

Figure 1 shows the distribution of reply times following each of the three mailings. Questionnaires are sent out in late September, with data due to be submitted to the central co-ordinating centre in mid-January, which effectively gives recipients of the survey three months in which to respond. As can be seen, the vast majority of questionnaires are returned within three weeks of the questionnaire or reminder being sent out. To an extent this is an artefact of the survey design, with the gaps between the first and second, and second and third mailings usually being 21 days. However, even responses to the final mailing, which could be received at any time until the end of the data collection window, have almost all been received by the end of three weeks.

Table 2 shows numbers of responses after each mailing and cumulative response rates. Of the 68,854 questionnaires for which return dates were recorded, 43,756 (63.5%) were received before the first reminder was sent, a further 11,850 (17.2%) were received after the first reminder (second mailing), and the remaining 13,248 (19.2%) arrived after the second reminder (third mailing). This corresponds to cumulative response rates of 32.8%, 41.7%, and 51.6% for the three response opportunities.

**Table 2:** Distribution of responses across three mailings

	**Replied after 1st mailing**	**Replied after 2nd mailing**	**Replied after 3rd mailing**	**Total**
Questionnaires returned	43756	11850	13248	68854
Cumulative response rate	32.8%	41.7%	51.6%	51.6%
**Overall** care: Excellent	20043	4958	4846	29847
	(47.2%)	(43.3%)	(37.9%)	
**Overall** care: Very good	14449	4073	4549	23071
	(34.0%)	(35.5%)	(35.6%)	
**Overall** care: Good, Fair *or* Poor	7950	2429	3374	13753
	(18.7%)	(21.2%)	(26.4%)	
**Cleanliness**: Very clean	28835	7572	7580	43987
	(66.7%)	(64.9%)	(58.4%)	
**Cleanliness**: Fairly clean	12880	3725	4781	21386
	(29.8%)	(31.9%)	(36.8%)	
**Cleanliness**: Not very clean *or* Not at all clean	1504	363	621	2488
	(3.5%)	(3.1%)	(4.8%)	
Confidence in **doctors**: Always	35465	9367	9845	54677
	(82.1%)	(80.5%)	(75.8%)	
Confidence in **doctors**: Sometimes	6517	1905	2583	11005
	(15.1%)	(16.4%)	(19.9%)	
Confidence in **doctors**: No	1185	367	552	2104
	(2.7%)	(3.1%)	(4.2%)	
Confidence in **nurses**: Always	32703	8506	9156	50365
	(75.9%)	(73.2%)	(70.6%)	
Confidence in **nurses**: Sometimes	9112	2738	3271	15121
	(21.1%)	(23.5%)	(25.2%)	
Confidence in **nurses**: No	1288	380	535	2203
	(3.0%)	(3.3%)	(4.1%)	
**Call button** answered: 0 minutes	4124	1087	1135	6346
	(16.3%)	(15.9%)	(14.4%)	
**Call button** answered: 1-2 minutes	10081	2632	2848	15561
	(39.8%)	(38.5%)	(36.1%)	
**Call button** answered: 3-5 minutes	7167	1987	2319	11473
	(28.3%)	(29.0%)	(29.4%)	
**Call button** answered: Over 5 minutes	3615	1018	1451	6084
	(14.2%)	(14.9%)	(18.4%)	
**Call button** answered: Never	365	119	130	614
	(1.4%)	(1.7%)	(1.6%)	

Table 3 shows results of the ordinal logistic regressions, one regression for each of the five outcome variables described above. A negative regression parameter estimate implies a lower probability of the patient reporting a high level of satisfaction. In all five cases the results show that patient satisfaction is lower among people who responded to the survey after receiving the first reminder, and lower still among those who only responded after the second mailing. These differences are statistically significant at the 0.05 level. In all five cases, satisfaction increases with age and is higher among male patients and people who were not subject to an emergency admission. In three out of the five cases, satisfaction declines with the length of the patient’s stay in the hospital.

**Table 3:** Ordinal logistic regression estimates with robust standard errors

	**Overall**	**Cleanliness**	**Doctors**	**Nurses**	**Call button**
Second mailing^1^	-0.135*	-0.052	-0.070*	-0.108*	-0.042
	(0.024)	(0.027)	(0.028)	(0.028)	(0.031)
Third mailing^1^	-0.329*	-0.287*	-0.258*	-0.184*	-0.180*
	(0.021)	(0.022)	(0.025)	(0.025)	(0.027)
Emergency admission^2^	-0.516*	-0.402*	-0.925*	-0.347*	-0.458*
	(0.027)	(0.031)	(0.030)	(0.027)	(0.030)
Age 36-50^3^	0.352*	0.145*	0.301*	0.328*	0.206*
	(0.036)	(0.037)	(0.042)	(0.038)	(0.044)
Age 51-65	0.577*	0.311*	0.734*	0.600*	0.304*
	(0.036)	(0.035)	(0.040)	(0.036)	(0.041)
Age 66+	0.638*	0.596*	0.987*	0.874*	0.320*
	(0.038)	(0.039)	(0.040)	(0.039)	(0.041)
Log (length of stay)	0.003	0.012	-0.062*	-0.124*	-0.165*
	(0.011)	(0.011)	(0.013)	(0.010)	(0.012)
Male^4^	0.349*	0.151*	0.280*	0.396*	0.282*
	(0.019)	(0.021)	(0.021)	(0.020)	(0.025)
Constants					-4.37
	-1.06	-3.11	-3.38	-3.03	-1.78
	0.549	-0.419	-1.30	-0.665	-0.316
					1.59
Observations	64,512	65,590	65,522	65,451	38,675
Wald chi sq	1694.2	748.3	2687.5	1717.0	922.7

Notes: * *p* < .05.

^1^Reference category is First mailing.

^2^Reference category is Planned admission.

^3^Reference category is 16–35.

^4^Reference category is Female.

These results, then, are consistent with previous research that has shown that patient satisfaction is lower among patients who require one or two reminders to respond to a mail survey than it is among respondents who return their questionnaires immediately. To determine whether the differences are of substantive as well as statistical significance, we calculated the predicted probabilities of a respondent giving different answers on the questionnaire based on the regression results shown in Table 3. To calculate these probabilities we assumed that respondents were male, had an emergency admission, were 36–50 years of age, and had a hospital stay of 3.2 days (which is the mean in the sample). Predicted probabilities are shown in Table 4.

**Table 4:** Predicted probabilities of responses to the satisfaction question

a) Overall rating of care						
			**Excellent**	**Very good**	**Good, Fair** ***or*** **Poor**	**N**
First mailing			0.41	0.37	0.22	41,190
Second mailing			0.38	0.37	0.25	11,055
Third mailing			0.33	0.38	0.28	12,267
Non-respondents			(0.33)	(0.38)	(0.28)	68,849
b) Cleanliness of ward						
			**Very clean**	**Fairly clean**	**Not very clean** ***or*** **Not at all clean**	**N**
First mailing			0.58	0.37	0.05	41,904
Second mailing			0.57	0.38	0.05	11,229
Third mailing			0.51	0.43	0.06	12,457
c) Confidence and trust in doctors						
			**Always**	**Sometimes**	**No**	**N**
First mailing			0.71	0.24	0.05	41,861
Second mailing			0.69	0.25	0.05	11,204
Third mailing			0.65	0.28	0.06	12,457
d) Confidence and trust in nurses						
			**Always**	**Sometimes**	**No**	**N**
First mailing			0.71	0.25	0.04	41,804
Second mailing			0.69	0.27	0.04	11,202
Third mailing			0.67	0.28	0.04	12,445
e) Time to respond to call button						
	**0 minutes**	**1-2 minutes**	**3-5 minutes**	**More than 5 minutes**	**Never**	**N**
First mailing	0.15	0.39	0.29	0.15	0.01	24,556
Second mailing	0.14	0.38	0.30	0.16	0.01	6,555
Third mailing	0.13	0.37	0.31	0.17	0.02	7,564

The largest difference in predicted probabilities between those who respond without a reminder and those who respond after the second reminder is in the first table, representing the analysis of responses to the overall rating of care question. The predicted probability of rating care as ‘Excellent’ declines from 0.41 to 0.33, a decline of 19%. The largest part of this is accounted for by an increase in the predicted probability of rating care as less than ‘Very good’ from 0.22 to 0.28, an increase of 27%.

If we assumed that those patients who responded to the final mailing are similar to non-respondents, we could use the predicted probabilities of the answers they give to the questionnaire to obtain predictions as to what reported patient satisfaction would have been given a 100% response rate. If we assume that all the non-respondents had the same predicted probabilities as those obtained for the latest responders, then we obtain the predicted frequency of response to this question shown in Table 5. We can compare these percentages to those shown in Table 1, and can see that the implication is a rather lower level of overall satisfaction: 21% of respondents reported that there care was less than ‘Very Good’, while 25% would have responded in this way if we assume non-respondents are like the latest actual respondents.

**Table 5:** Observed and predicted frequencies of response to the overall rating question

	**Excellent**	**Very good**	**Good, Fair or Poor**	**N**
First mailing	19,435	14,048	7,707	41,190
Second mailing	4,765	3,935	2,355	11,055
Third mailing	4,647	4,367	3,253	12,267
Non-respondents (predicted)	(22,989)	(26,293)	(19,567)	68,849
Overall percentage	39%	36%	25%	

Differences in the other predicted probabilities shown in Table 4, while still noticeable, are not as large. For example, the predicted probability of always having trust in doctors drops from 0.71 to 0.65, a decline of eight per cent, while the equivalent decline for nurses is six per cent.

## Discussion

The necessity for repeat mailings may be questioned on economic grounds, or out of concern not to harass patients. This issue is sometimes raised, for example, in discussions with NHS hospitals contracting for the annual survey (personal communication with an authorised contractor for the NHS inpatient survey), or by ethics committees when reviewing a research proposal that includes the patient experience survey as a data collection instrument. However, this paper shows that there is a relationship between a patient’s overall evaluation of their care and whether they are responding to the initial mailing or to a reminder. Less satisfied patients are less likely to respond to the initial mailing, but significant numbers of them do respond to reminders. This demonstrates that repeat mailings reduce response bias in patient surveys. Without the repeat mailings, the proportion of people reporting their care was Excellent or Very Good would be significantly higher. This study suggests that both patience—giving patients time to respond, and persistence—sending reminders, is required to ensure that the survey data do not exclude patients who have had a more negative experience of care. The wider implication of this paper is that bias could be introduced through small changes to the survey protocol. As health care systems become more and more dependent on patients’ evaluations of their care it is essential that we work to produce data that gives a true picture of patients’ experiences, rather than data that are misleading. In a paper titled “25 Years of Health Surveys: Does more data mean better data?”, Berk, Schur and Feldman [25] reflected that, in the US “…survey designers are the victims of their own success; as policy makers understand the value of survey data in assessing policy changes, growing demands for data force agency budgets to emphasize short-term efforts while postponing longer term investments in data quality”. One of their main recommendations is that more be invested in research on survey methods.

We might ask whether response rates could be increased further by sending more reminders and/or by extending the data collection period. We have already alluded to the potential ethical concerns that would arise from sending more reminders, to which we would have to add the fact that still more time would have elapsed from the actual inpatient experience to the completion of a questionnaire. In the case of the NHS survey of inpatient experience this currently means that most of the survey patients are discharged around June, and data collection ends the following January. Many of the final reminders in this survey are dispatched relatively late in the data collection period, effectively giving respondents little more than a month to respond. Although the majority of people who intend to respond will have done so in this time period, about 20 percent of people who responded after receipt of the second reminder took more than a month to do so. On balance, it would seem preferable to ensure that there is a period of two months from dispatch of the final reminder before the close of data collection, but further extension would probably not result in a great increase in responses, Figure 1 shows that the rate of responses does decline markedly after three weeks.

Non-response bias is not the only potential problem that we face in obtaining valid estimates of patient satisfaction. For example, post-discharge mail surveys may be superior to methods that involve questioning patients in hospital in that the more impersonal, anonymous nature of the data collection method may encourage more negative feedback. They may also be felt to be less intrusive by patients than methods involving face-to-face or telephone contact with researchers. On the other hand, it is possible that mail survey questionnaires are completed by someone other than the actual patient and such responses may differ from those that would have been given by the patients themselves [26].

One possible area for future research would be the extent to which the most reluctant responders to these questionnaires could be used as proxies for non-responders. Further information about their similarities and differences could lead to the development of non-response weight. We have shown that if we were to assume that non-responders were indeed similar to patients responding to the final reminder, then the change in estimated levels of satisfaction would be noticeable, but not substantial. However, it is conceivable that levels of satisfaction among non-responders are much lower than even the late responders, which would seriously undermine the validity of the data. The fact that we found a consistent relationship—satisfaction declining with the number of reminders—suggests that in this case the assumption that non-responders are similar to late responders may not be unreasonable, but further research in this area would be very useful, particularly given the importance of this survey in monitoring standards in the NHS. If it is shown that non-responders are similar to late responders, then we can more confidently claim that the method by which this survey is currently conducted is an effective way of obtaining reasonable estimates of patient satisfaction with care.

## Conclusions

We set out to investigate the importance of reminders in relation to the national (England) inpatient survey and found that late responders and those whose questionnaires were received after reminders had been sent were significantly less satisfied than those who responded to the initial mailing. We conclude that reminders have a significant and important effect, and that the current practice for the national surveys of sending two reminders to non-responders is appropriate and proportionate to the benefits of reducing non-response bias.

## Competing interests

The authors declare that they have no competing financial interests. Rachel Reeves was involved in the development and management of the national patient surveys as an employee of the Picker Institute Europe from 2001 to 2005. As a freelance consultant she has given advice on the design and use of surveys to policy makers and managers in the NHS.

## Authors’ contributions

DNB: designed the study, conducted the data analysis, interpreted the findings, drafted the paper and purchased the data from the Picker Institute Europe. EW: initiated the paper, devised the research questions, contributed to the interpretation of the findings, drafted, finalised and submitted the manuscript. RR: contributed to the conception of the study, interpretation of findings and drafting of the manuscript. DH: contributed to the research design, interpretation of findings and drafting the manuscript. All 4 authors gave consent for the final document to be submitted. All authors read and approved the final manuscript.

## Pre-publication history

The pre-publication history for this paper can be accessed here:

http://www.biomedcentral.com/1472-6963/14/153/prepub
